# DNA barcodes are ineffective for species identification of *Acropora* corals from the aquarium trade

**DOI:** 10.3897/BDJ.12.e125914

**Published:** 2024-07-17

**Authors:** Z. B. Randolph Quek, Zhi Ting Yip, Sudhanshi S. Jain, Hui Xian Vanessa Wong, Zayin Tan, Adrielle Ruth Joseph, Danwei Huang

**Affiliations:** 1 Department of Biological Sciences, National University of Singapore, Singapore, Singapore Department of Biological Sciences, National University of Singapore Singapore Singapore; 2 Yale-NUS College, National University of Singapore, Singapore, Singapore Yale-NUS College, National University of Singapore Singapore Singapore; 3 NUS High School of Math and Science, Singapore, Singapore NUS High School of Math and Science Singapore Singapore; 4 Lee Kong Chian Natural History Museum, National University of Singapore, Singapore, Singapore Lee Kong Chian Natural History Museum, National University of Singapore Singapore Singapore

**Keywords:** CITES, conservation, DNA barcoding, endangered species, mitochondrial genome, Scleractinia, stony corals

## Abstract

Species identification of stony corals (Scleractinia), which are regulated under the Convention on International Trade in Endangered Species of Wild Fauna and Flora, is critical for effective control of harvest quotas, enforcement of trade regulations and species conservation in general. DNA barcoding has the potential to enhance species identification success, depending on the specific taxon concerned and genetic markers used. For *Acropora*, DNA barcoding, based on the mitochondrial putative control region (mtCR) and the nuclear *PaxC* intron (*PaxC*), has been commonly used for species identification and delimitation, but the reliability and robustness of these loci remain contentious. Therefore, we sought to verify the applicability of this approach. In this study, we obtained 127 *Acropora* colonies from the aquarium trade to test the effectiveness of barcoding mtCR and *PaxC* for species identification. We were able to recover sequences for both loci in over half of the samples (n = 68), while gene amplification and sequencing of mtCR (n = 125) outperformed *PaxC* (n = 70). Amongst the 68 samples with both loci recovered, just a single sample could be unambiguously identified to species. Preliminary identities, based on only one gene, were assigned for 40 and 65 samples with mtCR and *PaxC*, respectively. Further analyses of 110 complete mitochondrial genomes obtained from GenBank showed that, despite the full length of the sequences, only eight species were delimited, of which only three species were correspondingly monophyletic. Therefore, we conclude that the commonly used DNA barcoding markers for *Acropora* are ineffective for accurate species assignments due to limited variability in both markers and even across the entire mitochondrial genome. Therefore, we propose that barcoding markers should generally not be the only means for identifying corals.

## Introduction

The trade of all stony corals (Cnidaria, Hexacorallia, Scleractinia) is regulated under Appendix II of the Convention on International Trade in Endangered Species of Wild Fauna and Flora (CITES). Specimens regulated include, but are not limited to, live samples and dead ornamental pieces. All traded corals should ideally be identified to species, allowing for the establishment of export quotas of threatened species, traceability and to enhance conservation efforts (Cohen et al. 2013). Recently, CITES proposed revisions to definitions pertaining to “*Trade in stony corals*”, acknowledging the difficulties in species-level identification in stony corals due to nomenclatural inconsistencies and identification challenges (CITES 2023). Specifically, consultation revolved around clarification of terminology, but, ultimately, all live or dead corals are to be identified at least to genus because of difficulties associated with species identification (CITES 2023; see also CITES (2013)). Indeed, visual identification of species can be confounded by morphological plasticity (Foster 1979, Todd 2008) and highly speciose groups like the genus *Acropora* are particularly challenging (CITES 2000, Bridge et al. 2023). Field identification of corals is problematic for regulation enforcement personnel who are not expertly trained and the difficulties of *ex situ* identification may be exacerbated by the issues associated with traded coral pieces that might either be cultured or fragmented from another colony (CITES 2000, Cohen et al. 2013).

*Acropora* is one of the most diverse and geographically widespread reef-building corals (Wallace 1999, Wallace et al. 2012), which only adds to the difficulty of identifying species. While recent studies have strongly recommended a modern integrated taxonomic approach that combines morphological and genomic data to delimit and identify *Acropora* species (Cowman et al. 2020; see also Quek and Huang (2021)), the extraordinary diversity of the genus remains unchallenged (Bridge et al. 2023). Within the coral trade, *Acropora* ranks amongst the most popular taxon of choice amongst aquarists, fuelled by their fast growth and diversity of forms and colours (Knop and Moorhead 2012). In fact, the popularity of *Acropora* warranted its own entry in a recent technical report by the Food and Agriculture Organisation (FAO) of the United Nations, with some 19 million pieces traded between 2007 and 2016 (Pavitt et al. 2021).

The molecular revolution has transformed our understanding of coral systematics and resulted in extensive taxonomic revisions (Kitahara et al. 2016). Recent developments of new phylogenomic approaches capitalising on high-throughput sequencing (HTS) have generated an unprecedented volume of data and further accelerated phylogenetic research on corals (Quek and Huang 2021). Nevertheless, due to the diversity within *Acropora*, it remains challenging to revise the clade, so numerous incongruences between its current morphological taxonomy and the phylogeny remain (Cowman et al. 2020, Bridge et al. 2023, Quek et al. 2023). It is also not feasible to monitor the identification accuracy of aquarium trade corals using HTS approaches on a regular basis due to time, cost and manpower limitations. Instead, traders utilise common names that might be region- or even country-specific, often relying inconsistently on growth form, colour and other morphological characteristics visible only in the aquarium setting, with no bearing on their valid scientific names. Therefore, until taxonomically consistent and visually identifiable characters are applied on traded *Acropora* corals, it is likely that misidentification of *Acropora* species will remain pervasive in the aquarium industry.

The importance of species identification cannot be overstated. Accurate species identification is important for conservation efforts. For example, overharvesting of rare corals can deplete local populations, leading to extirpations and downstream ecological consequences (reviewed in Dee et al. (2014)). On the regulatory front, CITES only requires all scleractinians to be minimally identified to genus, but the United States of America (USA), according to its Endangered Species Act, requires *Acropora* to be identified to species level (see Tlusty et al. (2023)). To enhance species identification in the aquarium trade, a recently proposed molecular framework for *Acropora* identification leverages two commonly used genes in *Acropora* phylogenetic studies — mitochondrial putative control region (mtCR) and nuclear *PaxC* intron (henceforth referred as *PaxC*) (Colin et al. 2022). Based on their workflow, they were able to assign species names to 44% and provisionally identify up to 80% of the 127 *Acropora* colonies tested.

Both mtCR and *PaxC* sequences are used widely in *Acropora* genetic studies, including investigation of species relatedness (Van Oppen et al. 2001, Van Oppen et al. 2003, Rosser 2015, Fukami et al. 2019). GenBank hosts > 1000 sequences of mtCR and > 400 sequences of *PaxC* from *Acropora* species (as of November 2023). Both markers span non-coding regions, therefore exhibiting a higher degree of sequence variability than coding genes. However, their suitability for species identification may be limited. Critically, the accuracy of deposited sequences is contingent on proper identification and curation of samples. Furthermore, the reliability and usability of either or both markers in *Acropora* remain contentious — while mtCR is able to delineate between genetically divergent clades (Suzuki et al. 2008, Fukami et al. 2021), the slow rate of evolution in mitochondrial DNA amongst scleractinians (Shearer et al. 2002) precludes fine-scale delimitation of all *Acropora* species (see also Van Oppen et al. (2001)). The *PaxC* intron alone may also not be ideal for species delimitation and identification. For example, non-monophyly of many *Acropora* species, based on *PaxC* sequences, was previously observed (Van Oppen et al. 2001, Rosser et al. 2017) Indeed, there is reason to suspect that species identification, based solely on these two markers, might be limited for *Acropora*.

In this study, we obtained 127 *Acropora* specimens from commercial aquarium vendors in Singapore in an attempt to identify them to species, following recent recommendations by Colin et al. (2022). Singapore has a bustling coral trade and corals are imported predominantly from Indonesia or Australia (data available at https://trade.cites.org/), making it ideal as a test-bed to investigate *Acropora* diversity in the aquarium trade. We sought to critically evaluate the applicability of DNA barcoding techniques on species identification from a diversity of *Acropora* species spanning different morphologies. Furthermore, to ascertain if the use of more molecular markers — apart from mtCR and *PaxC* — would improve species identification rates, we evaluated the potential for complete mitochondrial genomes to help delimit *Acropora* species. Our study is critical for determining if DNA barcoding can be broadly applied to recognise the diversity of *Acropora* corals in the aquarium trade, as well as understanding where improvements are needed for the enforcement of trade regulations.

## Material and methods

### Sampling strategy for traditional PCR and sequencing

We acquired 127 *Acropora* samples from commercial aquarium shops (n = 11) in Singapore. Where possible, we clarified the geographical origin of the fragments purchased. For most samples, small fragments which were common in the aquarium trade were obtained (Suppl. material 1). Common names and supposed species identity were also recorded when provided by vendors. To increase the diversity of *Acropora* sampled, we selected colonies from a variety of morphs and common names. Photographs of live colonies were taken and branch tip fragments of 2 cm were collected and stored in 100% ethanol at -80°C until further processing. Subsequently, each voucher specimen was bleached, dried and deposited at the Reef Ecology Laboratory, National University of Singapore (specimens available upon request).

### DNA extraction, amplification and sequencing

DNA extraction was performed using the DNeasy Blood and Tissue Kit (Qiagen) following the manufacturer’s protocol. Extracted DNA was then stored at −20°C until further processing. Markers as recommended in Colin et al. (2022) — mtCR and *PaxC* — were amplified using GoTaq Master Mix (Promega) following the manufacturer’s protocol. For mtCR gene amplification, the primers used were RNS2: 5′-CAG AGT AAG TCG TAA CAT AG-3′ and GR: 5′-AAT TCC GGT GTG TGT TCT CT-3′ (Suzuki et al. 2008) with a protocol of 94°C for 30 s, followed by 40 cycles at 94°C for 10 s, 56°C for 15 s and 72°C for 60 s, ending with a final phase of 72°C for 5 min. For *PaxC* gene amplification, primers used were FP1 5′-TCC AGA GCA TTA GAG ATG CTG G-3′ and RP1 5′-GGC GAT TTG AGA ACC AAA CCT GTA- 3′ (Van Oppen et al. 2001) with a protocol of 94°C for 30 s, followed by 31 cycles at 94°C for 10 s, 57°C for 30s and 72°C for 60 s, ending with a final phase of 72°C for 5 min.

Successful PCR amplification was visualised on a 1% agarose gel. PCR products were purified using SureClean (BioLine) and Sanger sequenced on an ABI 3730XL Genetic Analyser (Applied Biosystems). Raw sequences recovered were visualised and manually trimmed for quality, followed by *de novo* assembly using Geneious Prime® 2019.1.2. Sequences were checked with BLASTn (*megablast*) against the GenBank *nr* database to verify that the correct region was amplified.

### Species assignment using barcoding markers

We utilised a modified approach of species assignment from Colin et al. (2022) with the mtCR and *PaxC* sequence data. Importantly, we did not use the assignment of *Acropora* species groups as recent phylogenomics advances revealed that traditional species groups, based on morphology, are inconsistent with evolutionary genomics (Cowman et al. 2020, Bridge et al. 2023). Consequently, future major taxonomic revisions will likely require phylogenomic reconstructions to support the new systematics. In light of this, the primary purpose of DNA barcoding in this study is to determine the applicability of the method for accurate and consistent species identification, which has downstream implications for enforcement of trade regulations associated with the import of live *Acropora* corals. All cleaned sequences were searched with BLASTn (megablast) against the GenBank nucleotide collection (nr/nt) database. At the first cut, results were sorted by percentage identity. If there was a 100% match to only one species, the sample would be assigned that species for a marker. Otherwise, the results would be sorted by bit score, with the highest bit score prioritised as the species identity. However, if more than one species shared the same bit score, we then checked for percentage identity similarity and the highest percentage identity hit to a single species would be assigned as such. In the event multiple species shared the same percentage identity, no species would be assigned. This was conducted separately for each gene. If both markers concurred in their identification, the particular species identity would be assigned to the specimen.

### Evaluation of mitochondrial genomes for species delimitation

A total of 119 complete *Acropora* mitochondrial genomes were downloaded from GenBank. The mitochondrial genomes were analysed using a similar BLASTn workflow conducted for mtCR and *PaxC*. However, we removed the requirement of prioritising 100% percentage identity due to the length of mitochondrial genomes and, instead, sorted the results by bit score and finally the next highest percentage identity for species assignment. To ensure consistency across the genomes for a local BLASTn v.2.9.0, sequences were checked for mitochondrial rearrangements and subsequently rotated using MARS (Ayad and Pissis 2017) using the branch and bound method. The original entry would be disregarded for the purposes of species identification and any species with only one recorded specimen and sequence (n = 9) was omitted from analysis for comparison, but included as a possible hit result.

Apart from the BLASTn approach, we reconstructed a phylogeny using complete mitochondrial genomes, with four *Montipora* mitochondrial genomes as outgroups. Mitochondrial gene order was first verified to be consistent across all *Acropora* and *Montipora* samples. All sequences were then rotated as above and aligned using MAFFT-G-INS-i v.7.427 (Katoh and Standley 2013). Model selection was conducted using ModelTest-NG (Darriba et al. 2020) and a Maximum Likelihood phylogeny was reconstructed with RAxML-NG (Kozlov et al. 2019), based on 25 random and 25 maximum parsimony starting trees. We performed 500 bootstrap pseudoreplicates for node support estimation. The ML phylogeny was then piped into Bayesian implementation of the Poisson Tree Processes (bPTP) (Zhang et al. 2013) for species delimitation analysis.

## Results

### Sequencing and species assignment, based on mtCR and *PaxC*

**Figure 1.**
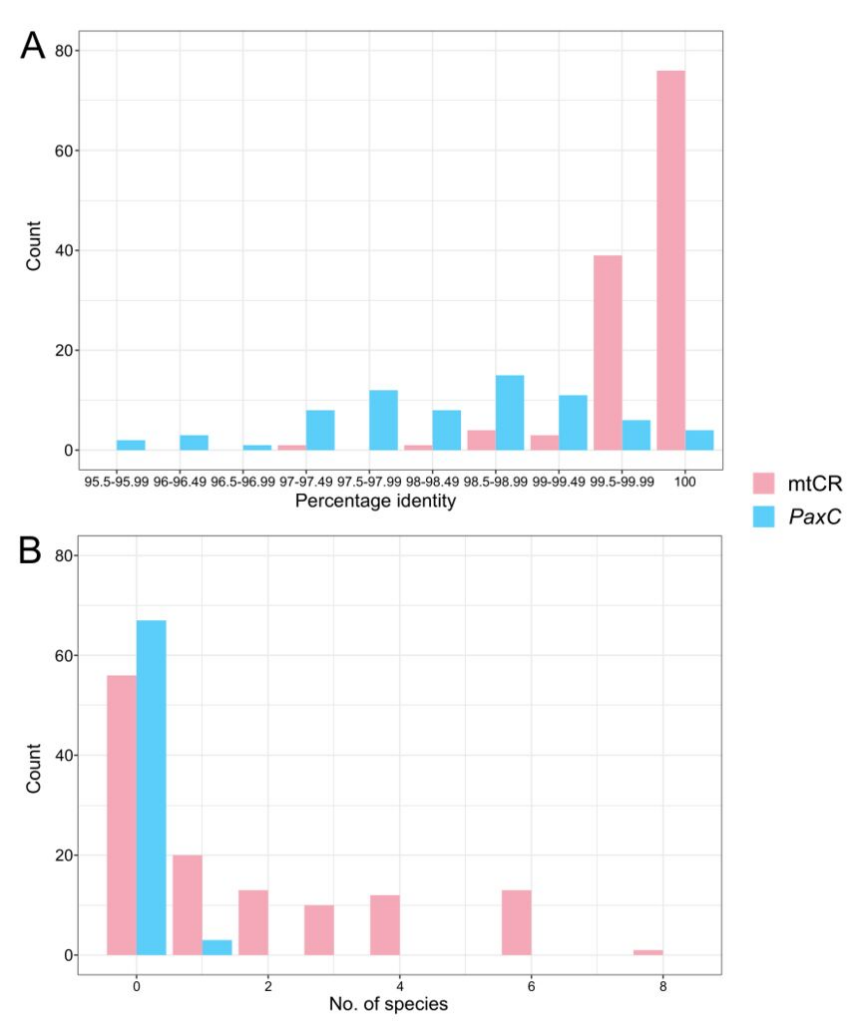
Frequency of sequences of each molecular marker according to (A) highest percentage identity of the queried sequence to the GenBank database and (B) number of matched species with 100% identity between the queried sequence and the GenBank database.

Sequences for mtCR were recovered from 125 samples and *PaxC* from 70 specimens. All sequences generated were deposited at Zenodo (https://doi.org/10.5281/zenodo.12538519). Based on mtCR, 40 samples had a preliminary species assignment after excluding samples with no unambiguous species epithet (i.e. not assigned as sp. or had qualifiers “cf.” or “aff.”), of which only 37 samples had a percentage identity of over 99%. The mean percentage identity for sequences with an assigned species was 99.76% (± SD 0.53%). *PaxC* performed better, with 65 specimens preliminarily assigned a species identification after following the same exclusions in mtCR. However, the percentage identity inferred from BLASTn to the reference database sequences for those with an assigned identity ranged between 95.70% and 100%, with a mean of 98.43% (± SD 1.01%) (Fig. 1A). Amongst the 57 samples with only mtCR and no *PaxC* sequences, only 24 could be preliminarily assigned a species identity after accounting for two samples that matched to reference sequences without a clear species epithet.

Between the two loci, mtCR had more species sharing 100% percentage identity matches compared to *PaxC*, making it less likely to have a definitive match and subsequent species identity, based on a perfect identity match alone (Fig. 1B). Finally, only a single sample had a species assignment (i.e. *A.abrotanoides*), based on concurrence in identity for both *PaxC* and mtCR (Suppl. material 1), out of 68 samples with both loci recovered.

### Mitochondrial genomes in for species delimitation

**Figure 2.**
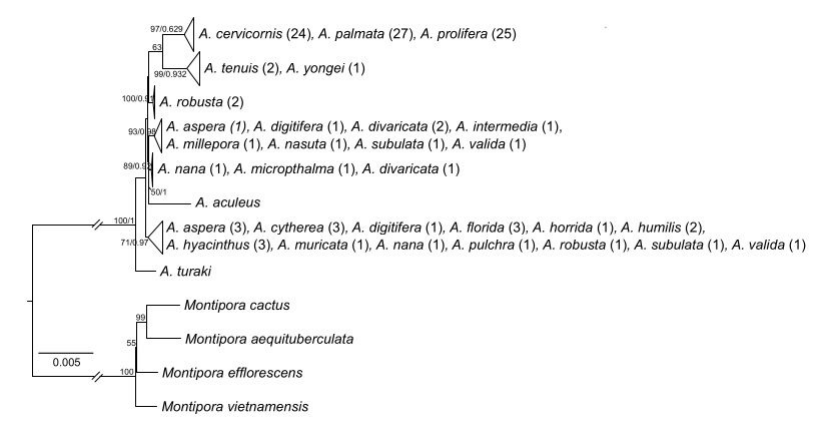
Maximum Likelihood phylogeny (RAxML-NG) of complete mitochondrial genomes of *Acropora*, with *Montipora* as an outgroup. Clades are collapsed, based on bPTP results for species delimitation and tips represent the sequenced-matched species identity with the number of samples denoted in parentheses. Numbers adjacent to nodes indicate bootstrap/posterior probability values from RAxML-NG and bPTP, respectively.

Complete mitochondrial genomes were neither able to accurately identify nor delimit species despite their length. After the removal of nine samples with a single record for the BLASTn analysis, only 35 out of 110 samples (31.82%) were correspondingly identified as the supposed species (Suppl. material 2). After excluding the Caribbean taxa (*A.cervicornis*, *A.palmata* and *A.prolifera*), only two species — *A.florida* and *A.robusta* — had an accurate hit for two out of the three samples in each. Out of a nominal 25 *Acropora* species used for phylogeny reconstruction, bPTP discerned only eight species, of which only three species had correspondingly one named species in the clade. Apart from the Caribbean clade (*A.palmata*, *A.prolifera* and *A.cervicornis*), all remaining species had PP > 0.9, based on bPTP. This generally corresponded with high bootstrap support (> 50) throughout the phylogeny (Fig. 2).

## Discussion

In this study, we sequenced barcodes for both mtCR and *PaxC* from 127 *Acropora* samples, for which we recovered mtCR sequences for 125 samples and 70 *PaxC* sequences. However, we were unable to provide a nominal species identity for all the samples other than *A.abrotanoides* with concurring results between both genes. These results suggest that both *PaxC* and mtCR are not useful for *Acropora* species identification and neither is the entire mitochondrial genome.

Databases are only as useful as the volume and accuracy of sequences and identities present within them. Coral identification is particularly difficult due to a lack of distinguishing characters in the skeleton, compounded by morphological plasticity (Todd 2008). Recent phylogenomic studies in *Acropora* have confirmed the complexity of the clade and the lack of consistency in determining species groups (*sensu* Wallace (1999)), based on morphological features. Indeed, the current evolutionary hypothesis in *Acropora* reveals trajectories inconsistent with traditional species groups delimited by morphology (Cowman et al. 2020, Bridge et al. 2023; see also Quek et al. (2023)). Most likely, current morphological characters used may not be sufficient to delimit species within *Acropora* (but see Ramírez-Portilla et al. (2022)). Furthermore, it is highly likely that many of the entries in GenBank might contain misidentifications considering the complexity of the clade, which reduces the efficacy of barcodes for species identification (Groenenberg et al. 2011). This is confirmed by our results, whereby only one sample was unambiguously assigned a species identification (i.e. *A.abrotanoides*), based on both mtCR and *PaxC*. Studies over the years have come up short when using mtCR and *PaxC* for species delimitation in *Acropora*, although they may be helpful in supporting lineages that are known to be phylogenomically distinct (Rosser et al. 2017, Ramírez-Portilla et al. 2022). We advise that neither of these two loci should be utilised for species identification without independent verification with alternative approaches, such as morphological and nuclear phylogenomic analyses.

Genome skimming is an easy and cheap method used to recover entire mitochondrial genomes in scleractinians (Quek and Huang 2021). However, in *Acropora*, we observe that variation across the entire mitochondrial genome is also highly limited, as evidenced by the short branch lengths and the inability of bit-score sorting to accurately classify species (Fig. 1, Fig. 2, Suppl. material 2). Comparing between phylogeny reconstruction and BLASTn, it appears a phylogeny reconstruction might be more useful for some species such as *A.robusta*. In this instance, considering the evolutionary distance between the monophyletic *A.robusta* clade comprising two samples (Fig. 1), the third *A.robusta* identified as *A.florida* (Fig. 2, Suppl. material 2) could possibly be a misidentification. Nevertheless, both are generally not sufficiently informative for accurate and consistent species identification. Mitochondrial evolution in scleractinians is notoriously slow and phylogenetically uninformative (Shearer et al. 2002, Hellberg 2006, Huang et al. 2008, Pratlong et al. 2017). Taken together, *PaxC*, mtCR and even complete mitochondrial genomes are unreliable for species identification of *Acropora* corals from the aquarium trade.

Fortunately, despite having hundreds of nominal species, not all *Acropora* species are common in the aquarium trade. Over the course of this study, our personal communications with aquarium vendors found that certain morphospecies tend to be traded more typically (e.g. *A.millepora*, *A.microclados*, *A.tenuis* and *A.spathulata*) and corals are also obtained from a limited set of localities (see also https://trade.cites.org/). Critically, users in the coral trade usually use names that are not rooted in scientific nomenclature, such as “Oregon blue tort” (*A.tortuosa*), “strawberry shortcake” (*A.microclados*), “Bali green slimer” (*A.yongei*) or “Tricolor valida” (*A.valida*), amongst others (Suppl. material 1). Moving forward, it would be worthwhile building a curated inventory of common names and species names that are verified by phylogenomic data and experts on coral taxonomy. This is especially useful in cultured corals, as only the parent colony needs to be identified to species. Subsequently, all stock has to be declared appropriately, with any deviations from the list to be reported with origin, photographs and finally, an identity assigned by an expert, ideally supported or at least periodically verified by phylogenomic analysis. As taxonomic revisions arise (e.g. Bridge et al. (2023)), this list can be updated accordingly. If an unknown *Acropora* colony is harvested from the wild and is of interest to regulators, nuclear phylogenomics would be required to accurately identify it to species (but see Ramírez-Portilla et al. (2022)). Hybrid capture analysis may be the most feasible amongst available phylogenomic approaches given currently available resources (Cowman et al. 2020, Quek et al. 2023). However, this method relies on a rich set of genomic data for many species and will likely only become viable in the coming years with new datasets being produced (e.g. Cowman et al. (2020), Bridge et al. (2023)). In the meantime, *in silico* methods can augment available data beyond those recovered from hybrid capture alone (see Quek et al. (2023)).

Identification of traded flora and fauna should ideally be performed at the species level, as the data can provide precise information for understanding trends and tracking the global trade of threatened species. In particular, robust and reliable species-level identification empowers CITES parties to implement targeted conservation policies and measures to improve the enforcement of wildlife trade regulations. Unfortunately, for scleractinian corals, species-level identification accounts for a small proportion of identification and, even so, it is often discarded as unreliable and unusable for analysis due to the high margin of error (Green and Shirley 1999). In this study, we found many *Acropora* colonies harvested from the wild for sale in the aquarium trade without proper taxonomic identification. The IUCN Red List (https://www.iucnredlist.org/) for *Acropora* lists many species of traded *Acropora* corals that run the gamut of categories, in ascending order of priority: least concern (e.g. *A.valida*, *A.yongei*), near threatened (*A.digitifera*, *A.tenuis*), vulnerable (*A.aspera*, *A.jacquelineae*) to even endangered (e.g. *A.suharsonoi*). Therefore, the identification of traded corals only to genus for permitting and reporting purposes may limit the conservation outcomes of trade regulations, especially in alleviating the threats of exploitation and extinction.

To reduce overharvesting of corals from wild populations, particularly amongst species at greater risk of extinction, we recommend that, as far as possible, corals for the aquarium trade should be sourced from aquaculture and coral farms instead. Furthermore, with the development of more accurate and rapid molecular methods to generate and analyse coral phylogenomic data in the future, as is currently occurring for other marine taxa (e.g. But et al. (2020), Naaum et al. (2021)), more effective regulatory and trade enforcement policies should be established once reliable species identities can be obtained for corals. Meanwhile, however, we emphasise that the two most popular *Acropora* barcoding markers — mtCR and *PaxC* — are inadequate for accurate species identification and more suitable loci need to be developed and tested.

## Conclusions

There is a pressing need to move towards a working model for the identification and traceability of traded scleractinian to boost conservation outcomes. The current CITES requirement with respect to corals allows declaration of species where possible, although genus identification is accepted. This is largely due to practical limitations and trade data can be adulterated where there is disregard for accurate species identification or lack of expertise in taxonomic identification. Furthermore, DNA barcodes are extremely limited in utility insofar as *Acropora* is concerned and should, therefore, not be the only means for species identification (but see Hoeksema and Arrigoni (2020)). To this end, we propose the following steps to improve traceability of the coral trade: (1) establish an inventory of the commonly traded corals with information on IUCN Red List status, source country, common name and scientific name, of which the specific species identified is to be verified by coral taxonomists and/or using nuclear phylogenomic analyses (at least until suitable barcodes for *Acropora* species identification are available), with the list updated in tandem with taxonomic revisions and conservation status updates; (2) all imports and exports to be declared against the list and any deviations from the list shall be appropriately recorded; (3) in the event of a deviation, images and a tissue sample shall be taken and retained by enforcement bodies for future verification; (4) periodic audits to be conducted, with stipulated identities verified by coral taxonomic expertise; (5) uncertain samples should be sent for nuclear phylogenomic analysis to ascertain their identities more accurately. Importantly, we recognise that some of these suggestions may not be feasibly adopted given the limited resources. These proposals are available as considerations for future conservation efforts and can be implemented in parts. Finally, the import and export of threatened species should be avoided; instead, coral farms can be a good avenue to supply the demand for all taxa, whether common or rare, thereby reducing the burden on wild populations.

## Supplementary Material

EBAADF9F-94BD-5312-BF3E-AD1CC055C45B 10.3897/BDJ.12.e125914.suppl1 Supplementary material 1 Table S1 Data type Table for results from data generated from mtCR and PaxC Brief description This supplementary material contains the results for identification of *Acropora* samples purchased, based on two loci: mtCR and *PaxC*. File: oo_1087147.xlsx https://binary.pensoft.net/file/1087147 Quek ZBR, Yip ZT, Jain SS, Wong HXV, Tan Z, Joseph AR, Huang D

30F56751-5247-59D4-90C1-28CBA59CFF86 10.3897/BDJ.12.e125914.suppl2 Supplementary material 2 Table S2 Data type Table for results based on complete mitochondrial genomes Brief description This supplementary material contains the results for identification of *Acropora* samples purchased, based on complete mitochondrial genomes. File: oo_1087149.xlsx https://binary.pensoft.net/file/1087149 Quek ZBR, Yip ZT, Jain SS, Wong HXV, Tan Z, Joseph AR, Huang D

## References

[B11305206] Ayad L. A.K., Pissis S. P. (2017). MARS: improving multiple circular sequence alignment using refined sequences.. BMC Genomics.

[B11302155] Bridge T. C.L., Cowman P. F., Quattrini A. M., Bonito V. E., Sinniger F., Head C. E.I., Hung J. Y., Halafihi T., Rongo T., Baird A. H. (2023). *Acroporatenuis* relationship: traditional taxonomy obscures systematics and biogeography of the ‘*Acroporatenuis*’ (Scleractinia: Acroporidae) species complex.. Zoological Journal of the Linnean Society.

[B11305215] But G. W.C, Wu H. Y., Shao K. T., Shaw P. C (2020). Rapid detection of CITES-listed shark fin species by loop-mediated isothermal amplification assay with potential for field use.. Scientific Reports.

[B11302147] CITES (2000). Identification and reporting requirements for trade in specimens of hard coral..

[B11302099] CITES (2013). Trade in stony corals: List of coral taxa where identification to genus level is acceptable.. https://cites.org/eng/node/1274.

[B11302131] CITES (2023). Consultation on trade in stony corals. https://cites.org/eng/node/137000.

[B11302090] Cohen F. P. A., Valenti W. C., Calado Ricardo (2013). Traceability issues in the trade of marine ornamental species. Reviews in Fisheries Science.

[B11302270] Colin L., Abed-Navandi D., Conde D. A., Craggs J., Silva R. da, Janse M., Källström B., Pearce-Kelly A., Yesson C. (2022). What’s left in the tank? Identification of non-ascribed aquarium’s coral collections with DNA barcodes as part of an integrated diagnostic approach. Conservation Genetics Resources.

[B11302197] Cowman P. F., Quattrini A. M., Bridge T. C.L., Watkins-Colwell G. J., Fadli N., Grinblat M., Roberts T. E., McFadden C. S., Miller D. J., Baird A. H. (2020). An enhanced target-enrichment bait set for Hexacorallia provides phylogenomic resolution of the staghorn corals (Acroporidae) and close relatives. Molecular Phylogenetics and Evolution.

[B11305224] Darriba D., Posada D., Kozlov A. M., Stamatakis A., Morel B., Flouri T. (2020). ModelTest-NG: a new and scalable tool for the selection of DNA and protein evolutionary models.. Molecular Biology and Evolution.

[B11740383] Dee Laura E., Horii Stephanie S., Thornhill Daniel J. (2014). Conservation and management of ornamental coral reef wildlife: Successes, shortcomings, and future directions. Biological Conservation.

[B11783763] Foster Ann Budd (1979). Phenotypic plasticity in the reef corals Montastraea annularis (Ellis & Solander) and Siderastrea siderea (Ellis & Solander). Journal of Experimental Marine Biology and Ecology.

[B11302367] Fukami H., Iwao K., Kumagai N. H., Morita M., Isomura N. (2019). Maternal inheritance of F1 hybrid morphology and colony shape in the coral genus *Acropora*. PeerJ.

[B11302406] Fukami H., Niimura A., Nakamori T., Iryu Y. (2021). Species composition and mitochondrial molecular phylogeny of *Acropora* corals in Funakoshi, Amami-Oshima Island, Japan: A proposal for its new taxonomic grouping. Galaxea, Journal of Coral Reef Studies.

[B11305244] Green E., Shirley F. (1999). The global trade in coral..

[B11740436] Groenenberg Dick S. J., Neubert Eike, Gittenberger Edmund (2011). Reappraisal of the “Molecular phylogeny of Western Palaearctic Helicidae s.l. (Gastropoda: Stylommatophora)”: When poor science meets GenBank. Molecular Phylogenetics and Evolution.

[B11305252] Hellberg M. E. (2006). No variation and low synonymous substitution rates in coral mtDNA despite high nuclear variation.. BMC Evolutionary Biology.

[B11740445] Hoeksema Bert W., Arrigoni Roberto (2020). DNA barcoding of a stowaway reef coral in the international aquarium trade results in a new distribution record. Marine Biodiversity.

[B11305261] Huang D., Meier R., Todd P. A., Chou L. M. (2008). Slow mitochondrial COI sequence evolution at the base of the metazoan tree and its implications for DNA barcoding.. Journal of Molecular Evolution.

[B11305270] Katoh K., Standley D. M. (2013). MAFFT Multiple Sequence Alignment Software Version 7: improvements in performance and usability.. Molecular Biology and Evolution.

[B11783781] Kitahara Marcelo V., Fukami Hironobu, Benzoni Francesca, Huang Danwei (2016). The New Systematics of Scleractinia: Integrating Molecular and Morphological Evidence. The Cnidaria, Past, Present and Future.

[B11302239] Knop D., Moorhead J., Lucas J. S., Southgate P. C. (2012). Aquaculture: Farming aquatic animals and plants.

[B11305290] Kozlov A. M, Darriba D., Flouri T., Morel B., Stamatakis A. (2019). RAxML-NG: a fast, scalable and user-friendly tool for maximum likelihood phylogenetic inference.. Bioinformatics.

[B11305300] Naaum A. M., Cusa M., Singh M., Bleicher Z., Elliott C., Goodhead I. B., Sanchez J. A. (2021). Validation of FASTFISH-ID: A new commercial platform for rapid fish species authentication via universal closed-tube barcoding.. Food Research International.

[B11302260] Pavitt A., Malsch K., King E., Kachelriess D., Vannuccini S., Friedman K. (2021). CITES and the sea: Trade in commercially exploited CITES-listed marine species..

[B11305312] Pratlong M., Rancurel C., Pontarotti P., Aurelle D. (2017). Monophyly of Anthozoa (Cnidaria): why do nuclear and mitochondrial phylogenies disagree?.. Zoologica Scripta.

[B11302230] Quek Z. B. R., Huang D. (2021). Application of phylogenomic tools to unravel anthozoan evolution. Coral Reefs.

[B11302212] Quek Z. B. R, Jain S. S., Richards Z. T., Arrigoni R., Benzoni F., Hoeksema B. W., Carvajal J. I., Wilson N. G., Baird A. H., Kitahara M. V., Seiblitz I. G., Vaga C. F., Huang D. (2023). A hybrid-capture approach to reconstruct the phylogeny of Scleractinia (Cnidaria: Hexacorallia). Molecular Phylogenetics and Evolution.

[B11305321] Ramírez-Portilla C., Baird A. H., Cowman P. F., Quattrini A. M., Harii S., Sinniger F., Flot J. F. (2022). Solving the coral species delimitation conundrum.. Systematic Biology.

[B11302358] Rosser N. L. (2015). Asynchronous spawning in sympatric populations of a hard coral reveals cryptic species and ancient genetic lineages. Molecular Ecology.

[B11302473] Rosser N. L., Thomas L., Stankowski S., Richards Z. T., Kennington W. J., Johnson M. S. (2017). Phylogenomics provides new insight into evolutionary relationships and genealogical discordance in the reef-building coral genus *Acropora*. Proceedings of the Royal Society B: Biological Sciences.

[B11302445] Shearer T. L., Van Oppen M. J. H., Romano S. L., Wörheide G. (2002). Slow mitochondrial DNA sequence evolution in the Anthozoa (Cnidaria). Molecular Ecology.

[B11302397] Suzuki G, Hayashibara T., Shirayama Y., Fukami H. (2008). Evidence of species-specific habitat selectivity of *Acropora* corals based on identification of new recruits by two molecular markers. Marine Ecology Progress Series.

[B11740392] Tlusty Michael F., Cawthorn Donna-Mareè, Goodman Orion L. B., Rhyne Andrew L., Roberts David L. (2023). Real-time automated species level detection of trade document systems to reduce illegal wildlife trade and improve data quality. Biological Conservation.

[B11783772] Todd Peter A. (2008). Morphological plasticity in scleractinian corals. Biological Reviews.

[B11305333] Todd P. A. (2008). Morphological plasticity in scleractinian corals.. Biological Reviews.

[B11302293] Van Oppen M. J. H., B J. McDonald, Willis B., Miller D. J. (2001). The evolutionary history of the coral genus *Acropora* (Scleractinia, Cnidaria) based on a mitochondrial and a nuclear marker: reticulation, incomplete lineage sorting, or morphological convergence?. Molecular Biology and Evolution.

[B11302284] Van Oppen M. J. H., Willis B. L., Van Vugt H. W. J., Miller D. J. (2003). Examination of species boundaries in the *Acroporacervicornis* group (Scleractinia, Cnidaria) using nuclear DNA sequence analyses. Molecular Ecology.

[B11302172] Wallace C. C. (1999). Staghorn corals of the World: A revision of the genus *Acropora*.

[B11302188] Wallace C. C., Done B. J., Muir P. R. (2012). Revision and catalogue of worldwide staghorn corals *Acropora* and *Isopora* (Scleractinia: Acroporidae) in the Museum of Tropical Queensland. Memoirs of the Queensland Museum.

[B11305342] Zhang J., Kapli P., Pavlidis P., Stamatakis A. (2013). A general species delimitation method with applications to phylogenetic placements.. Bioinformatics.

